# Military Service Roles and ALS Among Veterans: A Matched Case–Control Study

**DOI:** 10.1002/acn3.70079

**Published:** 2025-05-19

**Authors:** Asaf Honig, Roy Dayan, Amir Knaani, Hagai Levine, Marc Gotkine

**Affiliations:** ^1^ Department of Neurology Soroka Medical Center Beer‐Sheva Israel; ^2^ Faculty of Health Sciences Ben‐Gurion University of the Negev Beer‐Sheva Israel; ^3^ Department of Neurology Faculty of Medicine, Hadassah Medical Organization, Hebrew University of Jerusalem Jerusalem Israel; ^4^ Geha Mental Health Center Petach‐Tikva Israel; ^5^ Medical Corps, Israeli Defense Forces Ramat Gan Israel; ^6^ Faculty of Medicine, Braun School of Public Health, Hadassah Medical Organization Hebrew University of Jerusalem Jerusalem Israel

**Keywords:** amyotrophic lateral sclerosis (ALS), exercise, military service, motor neuron disease (MND), veterans

## Abstract

While military service is an established risk factor for amyotrophic lateral sclerosis (ALS), it remains unclear whether this association is linked to combat. We conducted a matched case–control study comparing 191 ALS patients who were veterans of the Israeli Defense Forces (IDF) with known military service type and 1910 matched controls. The ALS group had higher rates of combat service (46.0% vs. 22.7%) and parachuting (10.5% vs. 1.1%) in comparison with controls (*p* < 0.001 for both). In a multivariate model, combat service was associated with ALS (odds ratio 2.49, confidence interval [1.49–4.16], *p* < 0.01). The higher prevalence of combat roles among ALS patients expands our understanding of military service factors that contribute to ALS risk.

## 1 Introduction

Military service is a risk factor for amyotrophic lateral sclerosis (ALS) [1, 2, 3]. The association could stem from military service itself or antecedent factors associated with ALS risk. Most studies were performed in countries without compulsory drafts; thus, any correlation with specific activities [3, 4, 5] or with predisposing physical fitness [1] may reflect self‐selection of those with higher ALS risk [6, 7, 8]. Indeed, in Denmark, where military service is mandatory—though lacking recent major armed conflicts—veterans did not have higher ALS incidences [9]. Some explanations, such as exposure to toxins [4, 10], trauma [1] and intense physical activity [1, 10] could be specific to combat training, while others, such as smoking [1], may be common in all soldiers.

Thus, unresolved questions remain: is this association specific to combat soldiers? Does participation in active conflict affect risk? Does the association reflect selection bias? Some of these questions can be addressed by studying the Israeli ALS population; service in the Israeli Defense Forces (IDF) is mandatory, and most combat soldiers have participated in active combat. Thus, we examined the military history of IDF veterans with ALS in comparison to non‐ALS control veterans, comparing service in combat and non‐combat roles.

## 2 Methods

This was a matched case–control study. We compared sociodemographic and military service characteristics between ALS patients who served in the IDF (ALS group) and an age‐ and sex‐matched randomly selected group of control veterans (Control group).

The study population consisted of patients with clinically probable or definite ALS, seen in the Hadassah ALS clinic between 2009 and 2016; this is a tertiary nationwide referral center. Information regarding clinical, demographic, and military history was prospectively collected from 426 consecutive patients. Of these, 191 reported being IDF veterans and could accurately describe their military profession. To eliminate recall bias, we independently validated the military history in all cases where this data was available within the IDF database—60/191 (31.4%) of participants—and found 100% concordance. People with ALS who served in other countries were not included in the ALS group.

For each ALS veteran, 10 age‐ and sex‐matched veterans were assigned (*n* = 1910), based on year of recruitment, age at recruitment, and sex. Controls fulfilling the matching criteria were selected randomly from within the IDF archives (SAS 7.1; Random Sample function).

Type of service was stratified according to degree of physical activity, with intensity stratified from highest (infantry combat) to lowest (desk job). In addition, we recorded country of birth, IDF medical profile, military parachuting, and years of formal education (Table 1).

**TABLE 1:** Comparison between ALS and control in demographic and military‐related characteristics.

Demographics	Control (*n* = 1910)	ALS (*n* = 191)	*p*
	*N* (%) or mean (SD)	*N* (%) or mean (SD)	
Sex (Male)	1540 (80.6%)	154 (80.6%)	0.99^c^
Birth‐continent			
North America	27 (1.4%)	4 (2.1%)	0.52^b^
South America	17 (0.9%)	2 (1.0%)	0.69^b^
West Europe	89 (4.7%)	13 (6.8%)	0.21^b^
East Europe	211 (11.3%)	32 (16.8%)	< 0.001^c^
Asia	170 (9.1%)	14 (7.3%)	0.59^b^
Africa	163 (8.7%)	31 (16.2%)	< 0.001^c^
Australia	5 (0.3%)	0 (0.0%)	1.00^b^
Israel	1192 (63.6%)	95 (49.7%)	< 0.001^c^
Birth			
Abroad	682 (36.4%)	96 (50.3%)	< 0.001^c^
Israel	1192 (63.6%)	95 (49.7%)	< 0.001^c^
Origin			
Non‐Eastern European	703 (40.3%)	94 (49.2%)	0.017^c^
Eastern European	1043 (59.7%)	97 (50.8%)	0.017^c^
Age (years)	64.4 ± 11.6	63.6 ± 11.4	0.50^a^
Education (years)	9.6 ± 2.3	14.3 ± 2.9	< 0.001^a^
*Military‐related characteristics*			
Job Group			
Infantry combat soldiers	186 (12.6%)	54 (28.9%)	< 0.001^c^
Non‐infantry combat soldiers	149 (10.1%)	32 (17.1%)	< 0.001^c^
Combat support	567 (38.5%)	38 (20.3%)	< 0.001^c^
Home front	572 (38.8%)	63 (33.7%)	0.41^b^
Job Group			
Combat soldiers	335 (22.7%)	86 (46.0%)	< 0.001^c^
Non‐combat soldiers	1139 (77.3%)	101 (54.0%)	< 0.001^c^
Parachuting training	21 (1.1%)	20 (10.5%)	< 0.001^c^
Medical fitness upon recruitment			
Low	191 (11.5%)	16 (9.8%)	0.500^c^
High	1469 (88.5%)	148 (90.2%)	0.500^c^
Medical fitness upon releasement			
Low	400 (23.0%)	30 (18.3%)	0.168^c^
High	1339 (77.0%)	134 (81.7%)	0.168^c^

^a^ Mann–Whitney *U* test.

^b^ Fisher's exact test.

^c^ Chi‐square test.

We estimated exposure to active conflict by implementing a conservative assumption that combat soldiers would be deployed during the 8 years following their initial compulsory draft date. Accordingly, at least 96.7% of the participants served during a major war, that is, where virtually all combat soldiers were deployed and participated in battle.

To assess the association with combat military service, a multivariate logistic regression (Tables S1–S3) was conducted. Comparisons between groups were conducted using chi‐square tests and Fisher's exact tests for categorical variables or Mann–Whitney *U* tests for continuous variables. SPSS ver. 21 and Matlab version 2024b were used for statistical analysis.

Both Hadassah Institutional Review Board and IDF Ethical Review Board approved this study (approval numbers 0633‐08‐HMO and 1420‐2014, respectively).

## 3 Results

Table 1 compares demographic characteristics of ALS and controls, using Chi‐square tests and Fisher's exact tests for categorical variables (e.g., sex) or Mann–Whitney *U* tests for continuous variables (e.g., age).

Among the ALS group, higher rates of infantry and non‐infantry combat soldiers were found than in controls (28.9% vs. 12.6% and 17.1% vs. 10.1%, *p* < 0.001). Higher rates of parachute training were found in the ALS group (10.5% vs. 1.1%, *p* < 0.001). ALS and control groups had similar rates of “full‐fitness” medical profile both at recruitment (90.2% vs. 88.5%, *p* = 0.50) and discharge (81.7% vs. 77.0%, *p* = 0.17) from service.

After adjusting for education, country of birth, and ethnicity in a multivariate logistic regression, combat military service was associated with ALS (odds ratio, OR = 2.49, 95% confidence interval, CI [1.49–4.16]; *p* < 0.01).

When sexes were analyzed separately, a multivariate model showed that combat military service was associated with ALS among females (Table S2b, OR = 22.17, 95% CI [5.34–92.06]; *p* < 0.01, classifying 94% of cases). Adjusting for sex did not significantly alter the other logistic regression analysis.

In an adjusted multivariate model for infantry soldiers, the ALS group had a trend towards more parachuting training that did not reach significance (Table S3, OR = 3.90, 95% CI [0.90–16.87], *p* = 0.07, classifying 91% of cases).

## 4 Discussion

We found a significant association between combat service and ALS, as demonstrated by both univariate and multivariate analyses (OR 2.49, CI [1.49–4.16], *p* < 0.01). Interestingly, we also found an independent association between female combat service and ALS (OR 22.17, CI [5.34–92.06], *p* < 0.01).

Our analysis suggests a relationship between the more intensive physical activity within military service and ALS. Several factors might explain this association. Firstly, the extreme physical activity experienced by combat soldiers, especially infantry soldiers, could be a causative factor, as suggested in previous postulations [1, 6, 7, 10]. The possible association between physical activity and ALS, however, is controversial [1]. In a nonmilitary setting, an inverse association between self‐reported physical activity and ALS has been observed [11]. It is possible that certain aspects of physical exertion within a military setting are unique; for example, the cognitive state accompanying strenuous exertion allows the individual to push themselves closer to their physical limitations, similar to competitive sports [7].

The mechanism underlying the association between strenuous physical activity and ALS has been hypothesized to be due to effects of gene transcription from the exercise itself [12] predisposing genetic makeup [8] or environmental lifestyle profile [13]. Another possibility is that repeated neuroaxis trauma, prevalent among combat soldiers [14], could contribute to ALS risk [1]. The association with trauma has been observed in both animal models and human epidemiological studies, although it remains a topic of debate [14]. This could also explain the higher prevalence of military parachuting training among ALS patients, as paratroopers are more likely to sustain trauma [15].

Our study population reduces the aspect of self‐selection bias and may accentuate the effects mediated by active combat, but has some limitations. The information regarding military roles only captures the period when mandatory military service was performed—3 years for men and 2 years for women. Although information beyond that period was not available, the majority of individuals likely performed reserve duty after this time, and some may have tended to maintain physical fitness, thus amplifying the effects of factors present during military service. Additionally, those in more physically demanding roles are more likely to perform more reserve duty, therefore increasing the exposure to constitutive factors. Furthermore, the study's retrospective nature also prevented the inclusion of veterans who died from ALS before the research commenced. Although military service in Israel is obligatory, entry into combat service, particularly elite units, is influenced by factors such as motivation, ideology, and education, which could serve as confounders.

The study classified veterans according to their level of combat activity. Nevertheless, categories remained broad, leaving room for variations in physical activity, exposure to active conflict, and injuries experienced by different combat soldiers. Future research would benefit from more personalized military histories.

Despite focusing on IDF soldiers, the study's findings could potentially contribute to a broader understanding of the association between military service and ALS prevalence. Nevertheless, the observations presented here only indicate an association, and further investigations are required to comprehend the implications fully.

In conclusion, the study highlights associations between military roles and ALS. Findings could direct further research on the pathogenesis of ALS, aiming for prevention and early detection.

## Author Contributions

The authors takes full responsibility for this article.

## Disclosure

The authors have nothing to report.

## Conflicts of Interest

The authors declare no conflicts of interest.

## Supporting information


Table S1.

## Data Availability

The data that support the findings of this study are available on request from the corresponding author. The data are not publicly available due to privacy or ethical restrictions.
