# Supplementary material for: Military Service Roles and ALS Among Veterans: A Matched Case–Control Study
Source: Ann Clin Transl Neurol. 2025 May 19;12(8):1702–5. doi: 10.1002/acn3.70079 (PMC12343322; doi:10.1002/acn3.70079)
Supplement: Supplementary file 1 — Table S1. [file ACN3-12-1702-s001.docx]

*Table 1S – Factors associated with ALS, Multivariate Logistic Regression*

|  | OR | 95% CI of OR | | P-value |
| --- | --- | --- | --- | --- |
|  |  |  |  |  |
| Education (y) | 3.00 | 2.52 | 3.56 | <.001 |
| Born in Israel | 0.45 | 0.28 | 0.74 | 0.002 |
| Eastern European Origin | 0.25 | 0.15 | 0.43 | <.001 |
| **Combat military service** | 2.49 | 1.49 | 4.16 | <.001 |

OR – odds ratio; CI – confidence interval.

*Table 2S – Factors associated with ALS, Multivariate Logistic Regression, stratified by sex*

|  |  | OR | 95% CI of OR | | p-value |
| --- | --- | --- | --- | --- | --- |
|  |  |  | Lower | Upper |  |
| **a. Males** | Education (y) | 2.99 | 2.49 | 3.60 | <.001 |
|  | Born in Israel | 0.49 | 0.28 | 0.86 | 0.012 |
|  | Eastern European Origin | 0.27 | 0.15 | 0.48 | <.001 |
|  | **Combat military service** | 1.61 | 0.91 | 2.84 | 0.103 |
| **b. Females** | Education (y) | 3.31 | 2.02 | 5.44 | <.001 |
|  | Born in Israel | 0.78 | 0.18 | 3.33 | 0.739 |
|  | Eastern European Origin | 0.16 | 0.04 | 0.63 | <.001 |
|  | **Combat military service** | 22.17 | 5.34 | 92.06 | <.001 |

OR – odds ratio; CI – confidence interval.

*Table 3S – Odds Ratio of Multivariate Logistic Regression for Predicting ALS among* *infantry soldiers based on* *parachuting training*

|  | OR | 95% CI of OR | | P-value |
| --- | --- | --- | --- | --- |
|  |  | Lower | Upper |  |
| Education (y) | 3.17 | 2.07 | 4.85 | <.001 |
| Born in Israel | 0.50 | 0.16 | 1.50 | 0.215 |
| Eastern European Origin | 0.13 | 0.04 | 0.44 | 0.001 |
| **Parachuting training** | 3.90 | 0.90 | 16.87 | 0.069 |

OR – odds ratio; CI – confidence interval.
